# Chemotaxing neutrophils enter alternate branches at capillary bifurcations

**DOI:** 10.1038/s41467-020-15476-6

**Published:** 2020-05-13

**Authors:** Xiao Wang, Mokarram Hossain, Ania Bogoslowski, Paul Kubes, Daniel Irimia

**Affiliations:** 1 BioMEMS Resource Center, Department of Surgery, Massachusetts General Hospital, Harvard Medical School, Shriners Burns Hospital, Boston, MA USA; 2 Department of Physiology and Biophysics, University of Calgary, Calgary, Alberta Canada

**Keywords:** Biological techniques, Biophysics, Immunology, Engineering

## Abstract

Upon tissue injury or microbial invasion, a large number of neutrophils converge from blood to the sites of injury or infection in a short time. The migration through a limited number of paths through tissues and capillary networks seems efficient and ‘traffic jams’ are generally avoided. However, the mechanisms that guide efficient trafficking of large numbers of neutrophils through capillary networks are not well understood. Here we show that pairs of neutrophils arriving closely one after another at capillary bifurcations migrate to alternating branches in vivo and in vitro. Perturbation of chemoattractant gradients and the increased hydraulic resistance induced by the first neutrophil in one branch biases the migration of the following neutrophil towards the other branch. These mechanisms guide neutrophils to efficiently navigate through capillary networks and outline the effect of inter-neutrophil interactions during migration on overall lymphocyte trafficking patterns in confined environments.

## Abstract (web summary)

Neutrophils mobilize rapidly and travel through the vasculature and microcirculation en masse in response to inflammatory stimuli. Here the authors use a microfluidic device and intravital microscopy to show neutrophils move through tissues in a highly ordered pattern, taking turns to file into alternate branches at bifurcations.

## Introduction

Neutrophils are the most abundant subpopulation of white blood cells in the blood circulation. They serve as the first line of host defense in tissue injury and infections. Upon tissue injury or microbial invasion, neutrophils rapidly migrate to the sites, eliminating microbes and mediating further immune responses^1,2^. This process is partly facilitated by chemotaxis—a process in which neutrophils migrate along the gradient of chemoattractant released by damaged tissue, microbes, or other leukocytes^2^. In vivo studies in the liver^3^, skin^4^, lymph node^5^, and lungs^6,7^ of animal models have shown that neutrophils navigate in a coordinated and uniform fashion toward targets, through tissues and capillary networks. However, the principle that governs the efficient trafficking of a group of neutrophils through capillary networks remain to be uncovered.

Here, we study the migration patterns of consecutive neutrophils through capillary branches in vivo and in vitro. In mouse models of liver and lymph node, we find that consecutive neutrophils moving through capillaries toward sites of infection and injury take alternative routes more often than predicted by random decisions. To explore the mechanisms that could explain this unexpected phenomenon, we employ microfluidic devices with branching channels. We find that consecutive neutrophils in vitro take alternating branches with even higher precision than observed in vivo. Enabled by the controlled microenvironment of these experiments, we uncover that neutrophils moving through small channels can obstruct the channels and bias the migration of follower neutrophils toward alternative routes by perturbing the chemoattractant gradients behind them and increasing the hydraulic resistance of the channels they are entering.

## Results

### Neutrophil migration bias at capillary bifurcations in vivo

**Fig. 1:**
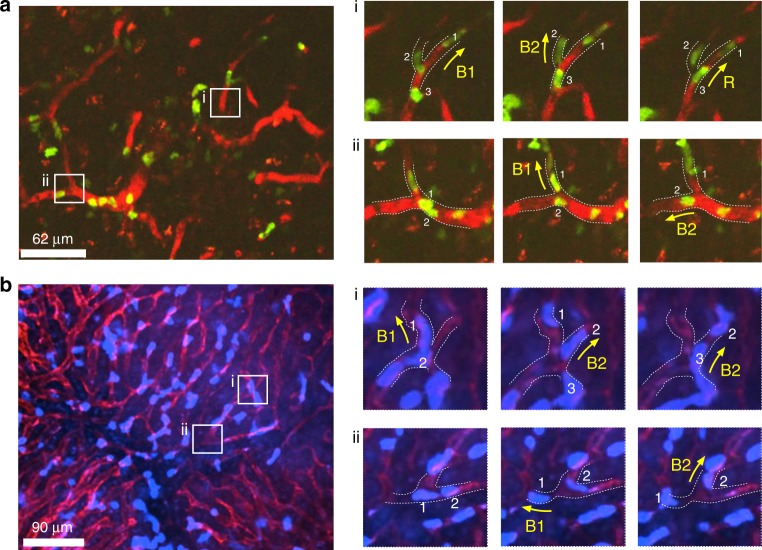
Consecutive decision making of neutrophil squads at capillary bifurcations in vivo. **a** Multiphoton-microscopic imaging of neutrophils moving in lymph node capillaries following the infection with *S. aureus*. **b** Multiphoton-microscopic imaging of neutrophils moving in liver capillary following local tissue damage. Panel i and ii are the zoom-in time-lapse images at the locations indicated with the white square. B1 and B2 represents branch 1 and branch 2. The yellow arrows indicate the migration direction of the neutrophils. The white dashed lines highlight the outline of the capillary bifurcation.

We employed multiphoton microscopy and spinning-disk confocal intravital microscopy to observe the trafficking of neutrophils in capillary networks towards sites of tissue damage in the liver (N = 4 mice). We also observed the trafficking of neutrophils toward sites of *Staphylococcus aureus* infection in the mouse lymph node (*N* = 8 mice) (Fig. 1, Supplementary Fig. 2, Supplementary Movie 1, Supplementary Movie 2, Supplementary Table 1). We focused on the capillary bifurcations where two neutrophils migrated consecutively. We defined these neutrophils as two-neutrophil squads. If neutrophils migrate randomly at the bifurcations, one would expect that 50% would of them enter different branches and 50% would enter the same branches. Surprisingly, we found that 23 out of 32 two-neutrophil squads diverged into different capillary branches rather than advancing in the same branch (Supplementary Table 1 and Supplementary Movies 1 and 2). The difference between the observed (72%) and expected (50%) frequencies is significant (*p* < 0.05, two-tailed test). The in vivo results also show that inside capillary branches with cross-section smaller than 50 µm^2^, the percentage of neutrophils entering alternate branches is 80% (*N* = 20 neutrophil squads). In capillary branches larger than 50 µm^2^ the percentage drops to 60% (*N* = 10 neutrophil squads, Supplementary Table 1). This data suggests that the divergence of two-neutrophil squads at capillary bifurcations may not be a random process. Instead, consecutive neutrophils most often enter alternative branches.

### Microfluidic bifurcations for studying migration bias

We employed a microfluidic device consisting of two-level bifurcations towards chemoattractant reservoirs to recapitulate this alternative migration pattern in vitro (Supplementary Movie 3). Strikingly, all neutrophils in neutrophil squads following a formylmethionine–leucyl–phenylalanine (fMLP) gradient sequentially migrated to the alternative branches at bifurcations. The observations were similar to the in vivo observations.

**Fig. 2:**
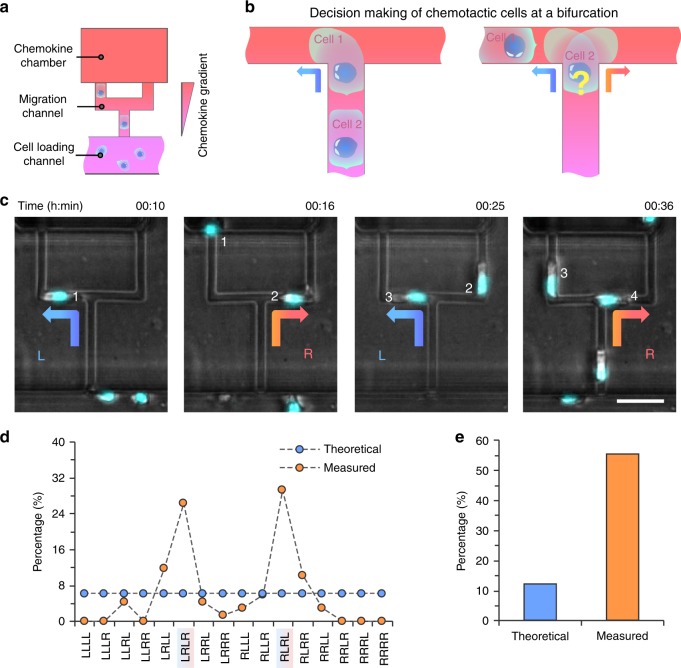
Consecutive decision making of neutrophil squads at microfluidic bifurcations in vitro. **a** A schematic of the microfluidic system for studying the sequential migration patterns of groups of chemotactic neutrophils at bifurcations. **b** A schematic showing consecutive neutrophils migrating alternatively into the left and the right branches. **c** Time-lapse bright-field microscopic images demonstrate that neutrophils in four-neutrophil squads enter alternate branches at bifurcations (left, right, left, right pattern). The scale bar is 25 µm. **d** Theoretical and measured percentages of the 16 possible directional patterns for 4-neutrophil squads passing through bifurcations (*N* = 4 donors, *N* = 68 4-neutrophil squads observed). **e** Theoretical and measured percentages of LRLR and RLRL combined.

To systematically study the directional decisions of groups of chemotactic neutrophils at bifurcations, we designed microfluidic chips that contain more than 200 simplified, microfluidic bifurcations units (Fig. 2). Each unit contains a two-branch bifurcation that starts with a cell-loading channel and is connected to a chemoattractant chamber. A chemoattractant gradient is established in each migration channel between the chemoattractant chamber (serving as chemoattractant reservoir) to the cell-loading channel (serving as sink), consistent with previously demonstrated principles^8^. Neutrophils are loaded in the cell-loading channel and migrate up the migration channel toward the chemoattractant chamber.

We characterized the gradient profile in the channels along each branch using fluorescein. The fluorescent intensity profile showed that the gradient was successfully established and maintained in both branches for more than 90 min (Supplementary Fig. 1a). The gradients in the left and right branches remained identical in the absence of neutrophils (Supplementary Fig. 1b). Chemotaxing neutrophils made one directional decision at the bifurcation (Fig. 2c). We quantified the direction of neutrophils at the bifurcation as Left (L) and Right (R), using the direction of migration relative to the glass bottom as spatial reference.

### Migration patterns of neutrophil squads at bifurcations

**Fig. 3:**
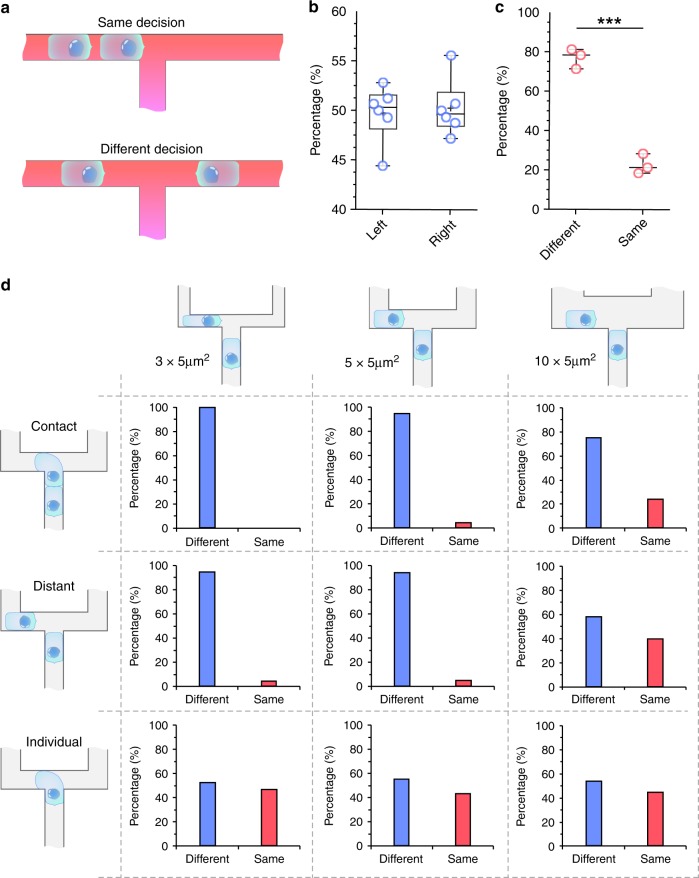
Directions of consecutive neutrophils at bifurcations with various cross-sections. **a** Schematic of two-neutrophil squads passing through bifurcations and entering the same or alternate branches. **b** The overall percentage of PMNs entering left or right branch (*N* = 6 donors, *N* = 447). Error bars the mean, median, IQR, min, and max. **c** The percentage of two neutrophils entering the same or opposite branch (*N* = 3 donors, *N* = 261). (***p < 0.001, unpaired, two-tailed *t* test). Error bars represent the standard deviation of measurements from three different donors. **d** The overall percentage of two consecutive neutrophils entering the same or opposite branches for a combination of 3 different channel cross-sections and three different intercellular distances. (*N* = 3 donors, *N* = 20 to 50 neutrophils for each condition).

We investigated the direction of successive neutrophils arriving at bifurcations in four-neutrophil squads (Fig. 2d). Theoretically, if arriving neutrophils enter branches at random, the directional patterns of 4 consecutive neutrophils passing through a bifurcation should be allotted equally in one of the 16 possible LR combinations and the percentage of each pattern would be equal to 6.25% (Fig. 2e, blue dots, *x*-axis). Strikingly, we observed that neutrophils in 4-cell “squads” enter alternate branches, along only two patterns: LRLR or RLRL (Fig. 2e). The total frequency of LRLR and RLRL patterns was ~55%, equal for the two patterns (*N* = 4 repeats, *N* = 68 four-neutrophil squads, Fig. 2e). The frequencies of LRLR and RLRL patterns were higher than all the other patterns combined (Fig. 2e). The frequencies of LLLL and RRRR patterns, when consecutive neutrophils followed each other into the same branch, were 0. In control experiments, we verified the symmetry of the system, by quantifying the frequency of the first neutrophil entering either one of the two migration channels at the bifurcation, in the absence of any following neutrophils. We found that the neutrophils enter the L and R branches with equal frequencies (*N* = 6, *N* = 447, Fig. 3b). Together, our observations show that neutrophils, when migrating closely in squads and arriving at bifurcations, do not enter the two branches randomly. Instead, they display alternate patterns.

### Neutrophil pairs enter alternate branches at bifurcations

To investigate the mechanisms that determine the alternate migration patterns of a group of neutrophils, we focused on the behavior of two-neutrophil squads (Fig. 3). We summarized the four possible combinations of neutrophil migration patterns into two states: same branch (LL and RR) and different branches (LR and RL) (Fig. 3a–c). We observed that the percentage of first neutrophils entering the left and right branches was equal (50 vs. 50%, *N* = 3, *N* = 261), indicating no bias for the first neutrophil entering the branches. However, the percentage of a second neutrophil entering a different branch is ~80%, significantly higher percentage than entering the same branch (*N* = 3, *N* = 261). This confirmed that neutrophils in two-cells squads are more likely to enter alternate branches than follow each other.

We investigated whether the cross-section of the bifurcation impacted the distribution of neutrophils from two-neutrophil squads at bifurcations (Fig. 3d). We designed the cross-section of the two branches after the bifurcation to be 15 µm^2^ (3 µm × 5 µm width × height), 25 µm^2^ (5 µm × 5 µm width × height) or 50 µm^2^ (10 µm × 5 µm width × height) (Fig. 3d, horizontal schematics). We maintained the cross-section before the bifurcation to 5 × 5 µm^2^ for all networks. We also categorized the intercellular distance between the two neutrophils into three categories, including contact, distant, and individual (Fig. 3d, vertical schematics). “Contact” represents the condition that the second neutrophil is in physical contact with the first neutrophil when arriving at the bifurcation. “Distant” indicates that when the second neutrophil arrives at the bifurcation, the first neutrophil is away from it, but still moving in one of the branches. “Individual” indicates neutrophils arriving at the bifurcation in the absence of other neutrophils in the migration channels. In the 3 × 5 and 5 × 5 µm^2^ bifurcations, we measured the percentage of neutrophils entering alternate branches to be 100% for “contact”, 95% for “distant”, and 50% for “individuals” (*N* = 3, *N* = 20 to 100). These results suggest that two neutrophils are more likely to enter alternative branches when they are moving closer to each other (Supplementary Fig. 3). The percentages sharply decrease in the 10 × 5 µm^2^ bifurcation, to 76% for contact, 60% for distant and do not change (~50%) for individuals (*N* = 3, *N* = 20 to 100). Our results show that in the symmetrical bifurcation, the chance of consecutive neutrophils entering alternate branches are correlated with the cross-section of the channels as well as the distance between the two neutrophils in the squad. The chances are higher in smaller bifurcation channels and when two neutrophils are closer.

Based on the observations, we hypothesize two mechanisms that impact migration patterns of chemotactic neutrophil squads arriving at bifurcations. The first mechanism involves the obstruction of small channels by the first neutrophils, which alters the hydraulic resistance of the channels. Subsequent neutrophils are sensitive to variations in pressure in front of them (barotaxis) and this mechanism could bias the neutrophils toward the lower hydraulic resistance path^9^. The second mechanism involves the alterations of the chemoattractant gradients in small channels by the moving neutrophils. The first neutrophils passing through a channel network could alter the chemoattractant gradient and can bias the migration of subsequent neutrophils toward the alternate branch, where the gradient is intact.

### Migrating neutrophils alter channel hydraulic resistance

**Fig. 4:**
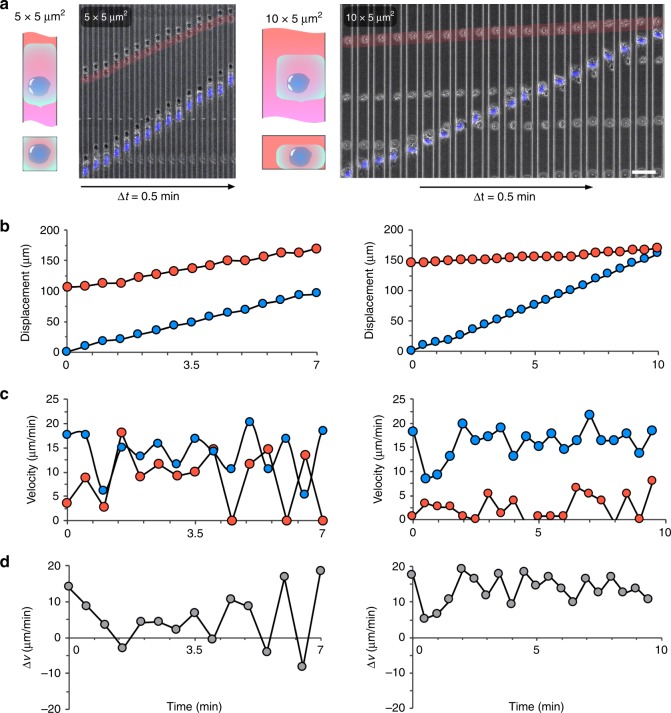
Neutrophils transiently alter the hydraulic resistance in small channels. **a** Bright-field microscopic time-lapse images showing the relative positions of migrating neutrophils and distant RBCs in 5 × 5 µm^2^ and 10 × 5 µm^2^ microchannels. The red transparent solid line indicates the trajectories of RBCs. The blue lines represent the neutrophils stained with Hoechst and pseudocolored blue. The scale bar is 20 µm. The time interval between two frames of images is 30 s. **b** Displacements over time for representative neutrophil–RBC pairs in 5 × 5 µm^2^ (left) and 10 × 5 µm^2^ (right) channels. **c** Velocity over time in the 5 × 5 µm^2^ (left) and 10 × 5 µm^2^ (right) channels. **d** The velocity difference of representative neutrophil–RBC pairs in 5 × 5 µm^2^ (left) and 10 × 5 µm^2^ (right) channels. In panels **b** and **c**, the red dots represent the RBCs and the blue dots represent the neutrophils.

**Fig. 5:**
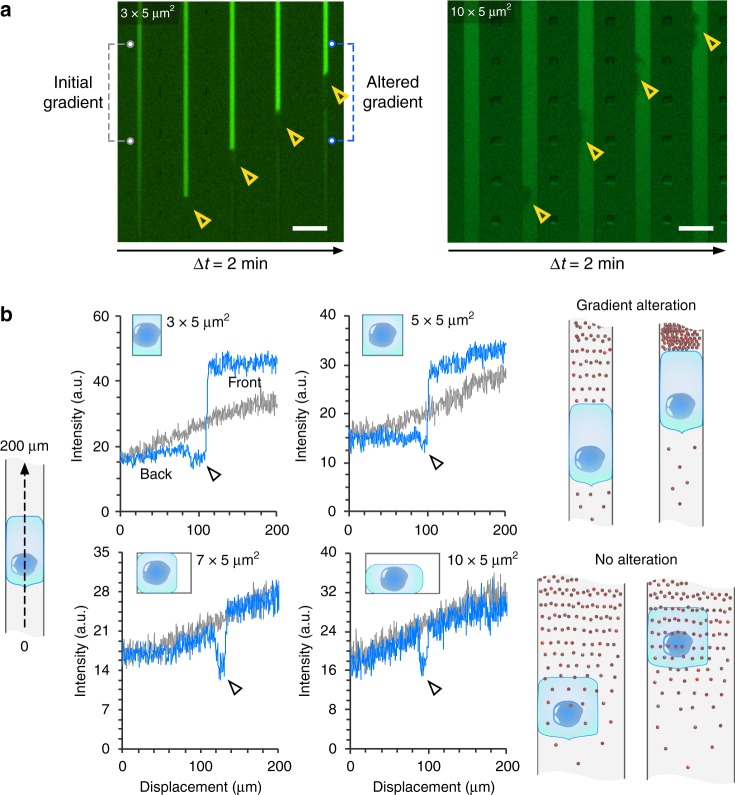
Neutrophils alter the chemoattractant gradients in small channels. **a** Fluorescence kymographs showing the alternation of chemical gradient in the 3 × 5 µm^2^, but not 10 × 5 µm^2^ channels. The yellow hollow arrows indicate the locations of one neutrophil at 2-min interval. The scale bar is 20 µm. **b** Measurements of fluorescent intensity profile across the neutrophils in channels with (blue solid lines) and without (gray solid lines) the neutrophil at various channel cross-sections. The black arrows indicate the location of the neutrophil. The rightmost schematics illustrate the concept of gradient alternation caused by moving neutrophils.

**Fig. 6:**
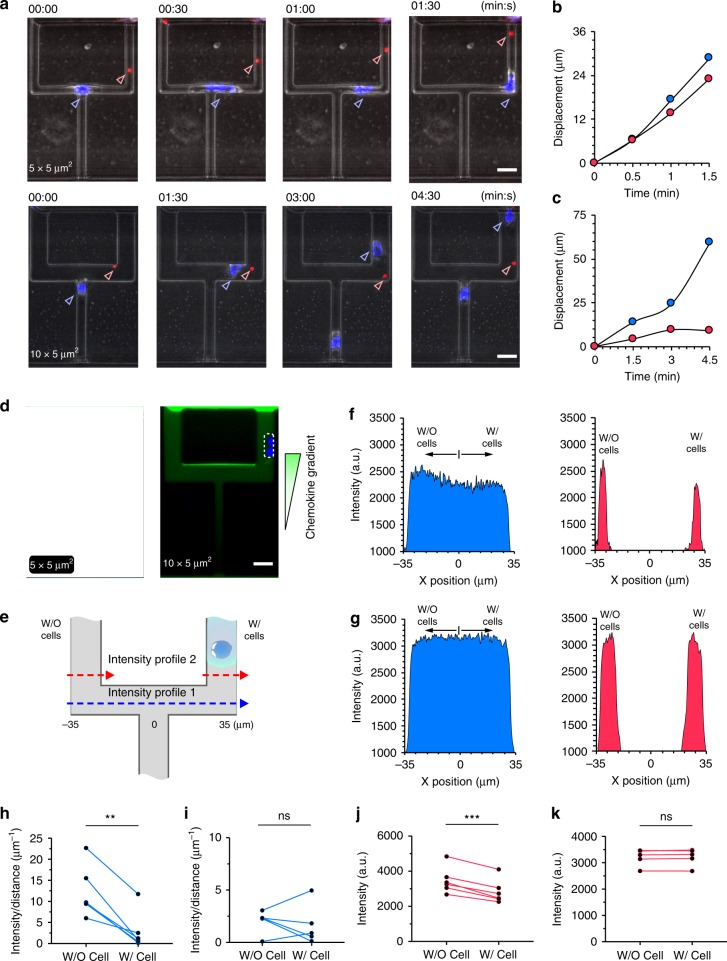
Neutrophil alters the hydraulic resistance and the chemoattractant gradient in the bifurcation. **a** Time-lapse microscopic images showing the migration of the neutrophils and the corresponding movement of the 2 µm diameter beads. The scale bar is 10 µm. The displacements of the neutrophil (blue) and the microbead (red) over time in 5 × 5 µm^2^ (**b**) and 10 × 5 µm^2^ (**c**) channels. **d** Representative fluorescent microscopic images show the chemical gradient in the bifurcation in the presence of a neutrophil. The white dashed rectangles indicate neutrophils. The scale bars are 10 µm. **e** A schematic showing the two measurement locations of the fluorescent intensity profiles. The fluorescent intensity profiles along the indicated lines in (**e**) in 5 × 5 µm^2^ (**f**) and 10 × 5 µm^2^ (**g**) channels. The blue and red areas correspond to profile 1 and 2. The fluorescence gradients in the two bifurcation branches without vs. with a neutrophil along profile 1 in 5 × 5 µm_2_ (**h**) and 10 × 5 µm^2^ (**i**) channels. The peak fluorescence intensity along profile 2 in the branches without vs. with a neutrophil in 5 × 5 µm^2^ (**j**) and 10 × 5 µm^2^ (**k**) channels. (**p < 0.01, ***p < 0.001, paired, two-tailed *t* test).

We tested the interplay between hydraulic and chemical signals in straight (Figs. 4 and 5) and bifurcating channels (Fig. 6). We employed a small number of fresh, human red blood cells (RBCs), preloaded in the channels during the priming steps, to determine if fluid is displaced in front of the moving neutrophils (Fig. 4a). In one typical example, inside a 5 × 5 µm^2^ channel, we observed that an RBC at 100 µm ahead of a neutrophil changed its position at an average velocity comparable to that of the moving neutrophil (Fig. 4b–d left panels). In one other example, in a 10 × 5 µm^2^ channel, an RBC at 150 µm ahead of a moving neutrophil, floated at 0–5 µm per min ahead of the neutrophil moving at ~20 µm per min, while other RBCs were by-passed by the moving neutrophil (Fig. 4b–d right panels). These situations suggest that fluid displacement occurs in front of neutrophils moving through the smallest channels.

### Migrating neutrophils alter chemoattractant gradients

To test whether neutrophils can alter the chemoattractant gradients, we spiked fluorescein in the fMLP solution and loaded it inside straight channels. Since fMLP and fluorescein have similar molecular weights (MW 437.55 g per mol and 332.31 g per mol, respectively), both molecules diffuse at a similar diffusion rate. The fluorescent intensity profile along the channel was used to indicate the profile of fMLP gradient. As Fig. 5a shows, as a neutrophil migrated in a 3 × 5 µm^2^ channel, the fluorescence intensity in front of it dramatically increased, much higher than the intensity without the neutrophil at the same location. The fluorescent intensity was not altered in 10 × 5 µm^2^ channels. We measured the fluorescent intensity profile with and without the presence of the neutrophil in various cross-sections (Fig. 5c). The results show that neutrophils alter the chemoattractant gradient in 3 × 5 µm^2^ and 5 × 5 µm^2^ channels. The fluorescence intensity is enriched in front of the neutrophil and decreased at the back, creating a much sharper gradient along the neutrophils than the initial gradient. In 7 × 5 µm^2^ and 10 × 5 µm^2^ channels, the fluorescence intensity profile remained the same, indicating that the chemoattractant gradient is not altered by the neutrophil.

### Neutrophils alter gradients and move fluid at bifurcations

Our experiments using straight channels suggest that chemotactic neutrophils can alter the chemoattractant gradient inside the channels and alter the hydraulic resistance of the channels. We applied this new knowledge to test the two proposed hypotheses regarding the mechanisms that govern the alternating paths of neutrophils moving through bifurcating channels (Fig. 6). To evaluate the fluid displacement in front of neutrophils moving through bifurcating channels, we loaded the channels sparsely with 2 µm TRITC-labeled, polystyrene microbeads. We tracked the migration of neutrophils and the relative displacement of microbeads (Fig. 6a–c). In 3 × 5 and 5 × 5 µm^2^ bifurcations, the microbeads moved forward as the neutrophils migrated into the same branch (Fig. 6a). The microbeads moved randomly in 10 × 5 µm^2^ channels, independent on the migration of the neutrophils. We measured the migration distance of the neutrophil and the microbead displacement over time (*N* = 5, Fig. 6b, c). In a typical example at the 5 × 5 µm^2^ bifurcation, a neutrophil migrated 28 µm in 1.5 min, while a microbead was displaced 23 µm (Fig. 6b). In another example, in the 10 × 5 µm^2^ bifurcation, the migration distance for a neutrophil (60 µm in 4.5 min) was larger than the bead displacement (9 µm, Fig. 6c). The neutrophil moving persistently up the branch eventually passes the bead. Taken together, the results in small and large bifurcating channels suggest that moving neutrophils displace the fluid through the channels ahead of them. This implies that neutrophils are relatively impermeable to fluid and could alter the hydraulic resistance of the channels inside which they reside.

We then measured the fluorescent intensity profiles in the presence of migrating neutrophils through bifurcations (Fig. 6d–g). The fluorescence intensity profiles were measured along the two lines as indicated in Fig. 6e. In the 5 × 5 µm^2^ channels, the presence of the chemotactic neutrophil in the right branch biased the chemoattractant gradient to the left (Fig. 6f). The fluorescent intensity behind the first neutrophil decreased, leading to asymmetrical gradient profiles in the two branches. Along profile 1, the gradient in the branch without the neutrophil is significantly steeper than the one with neutrophil (Fig. 6h, Supplementary Fig. 4). The peak fluorescence intensity is also significantly higher in the branch without the neutrophil than with the neutrophil (Fig. 6j). Consequently, the second neutrophil would experience steeper chemoattractant gradient in the alternating branch. In contrast, in the 10 × 5 µm^2^ channels, the fluorescent intensity profiles remained symmetrical in the two branches, despite the presence of the neutrophil (Fig. 6g, h, k). Our results indicate that migrating neutrophils through bifurcations alter the chemoattractant gradients in the two branches with a small cross-section. To test the contribution of hydraulic resistance alterations, we probed the migration of neutrophils through asymmetric bifurcations.

### Neutrophil pairs migrate through asymmetric bifurcations

**Fig. 7:**
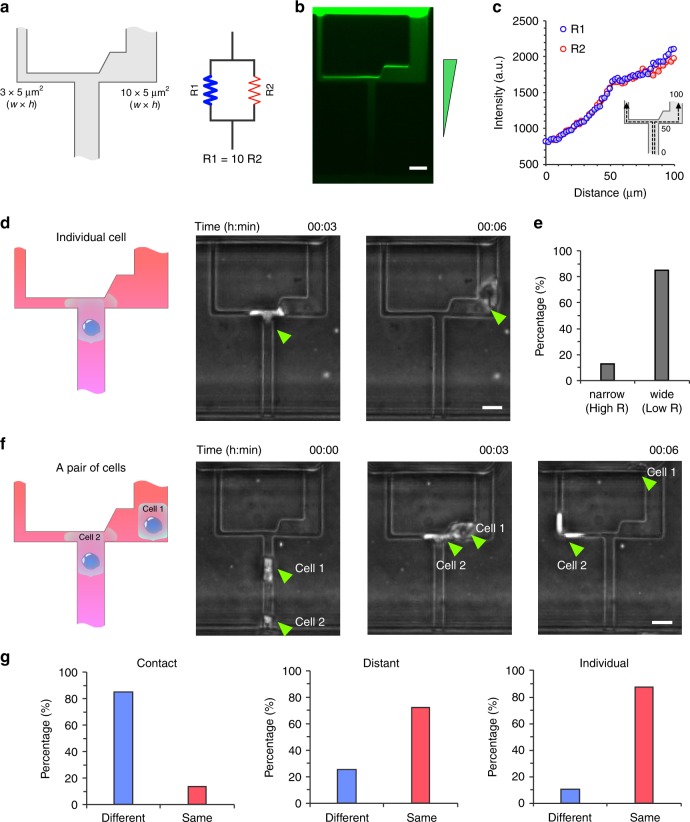
Migration patterns of consecutive neutrophils at asymmetric bifurcations. **a** The design of the microfluidic asymmetrical bifurcation and the corresponding electric circuit model showing the hydraulic resistance of the channels. R1 and R2 represent the hydraulic resistance of the narrow and wide channels, respectively. **b** A fluorescent microscopic image showing the fluorescent gradient at the bifurcation. The scale bar is 10 µm. **c** Measurements of the fluorescent intensity profiles along narrow (blue) and wide (red) channels. **d** A schematic illustration and experimental time-lapse images showing an individual cell passing through the asymmetric bifurcation. The green arrows indicate the cell. The scale bar is 10 µm. **e** A graph showing the percentage of cells entering the narrow and wide branches. (*N* = 3 donors, *N* = 181). **f** A schematic illustration and experimental time-lapse images showing the entrance of consecutive neutrophils from two-neutrophil squads in asymmetric branches at a bifurcation. The green arrows indicate the cells. The scale bar is 10 µm. **g** Graphs showing the percentage of neutrophil entering the same branch when migrating in two-neutrophil squads and in contact (*N* = 3 donors, *N* = 49) or separate (*N* = 3 donors, *N* = 31), or entering the channel individually (*N* = 3 donors, *N* = 69).

We explored the migration patterns of two-neutrophil squads at asymmetric bifurcations (Fig. 7a). The cross-sections of the branches are 3 × 5 µm^2^ and 10 × 5 µm^2^ and their length is equal. The hydraulic resistance *R* of the channels is different, estimated using the following equation^10^

1 $$R \approx \frac{{12\,\mu L}}{{wh^3\left( {1 - \frac{{0.63h}}{w}} \right)}},\,w\, > \, h,$$

where *w*, *h*, and *L* represent the width, height, and length of the channel and *µ* represents the viscosity of the fluid. The ratio of the hydraulic resistance of the narrow and wide channels is calculated to be ~10 (Fig. 7a). We measured the fluorescent intensity profiles along the narrow and wide branches, confirming that the chemoattractant gradients in the two channels are similar (Fig. 7b, c).

We monitored the trajectories of individual neutrophils at bifurcations. We found that 87% of the individual neutrophils migrated to the wide branch with lower hydraulic resistance (Fig. 7d, e). We also studied the migration pattern of neutrophils in two-neutrophil squads (Fig. 7f). We observed that while the first neutrophil most often entered the wider branch, the trajectory of the second neutrophil was dependent on the intercellular distance between the two neutrophils. When two neutrophils are closely following each other at the bifurcations (Fig. 7f, g), we observed that 86% of the time the second neutrophil entered the narrower branch. The percentage sharply decreases to 26% for “distant” conditions, and to 12% for “individuals” conditions (Fig. 7g). The results suggest that the impermeable neutrophils could alter the hydraulic resistance of small channels and is consistent with the barotaxis bias of neutrophils that prefer to migrate along the path with lower hydraulic resistance^9^.

### Migration of neutrophil armies through bifurcating networks

**Fig. 8:**
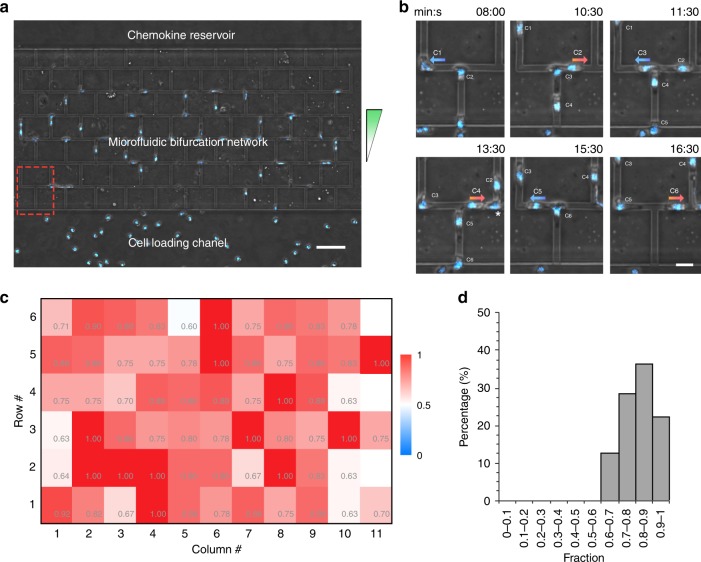
Migration patterns of an army of neutrophils in a microfluidic bifurcation network. **a** The design of the microfluidic bifurcation network. The scale bar is 50 µm. The green arrow indicates the chemoattractant gradient. The neutrophils were stained with Hoechst and pseudocolored blue. **b** Time-lapse microscopic images showing the migration patterns of six neutrophils at one of the bifurcations in the network. The location of the bifurcation is indicated with red dashed rectangle in panel (**a**). The scale bar is 10 µm. **c** A heatmap showing the fraction of neutrophils moving to alternate branches at all the bifurcations in the network (*N* = 3 donors, *N* = 993). The blue, white and red colors represent a fraction of 0, 0.5, and 1, respectively. **d** A histogram showing the distribution of the fractions derived from panel (c).

We explored the migration pattern of a large group of neutrophils in a microfluidic bifurcation network, which mimics complex microvascular networks in vivo. The device contains 63 bifurcations arranged into 11 columns and 6 rows (Fig. 8a). The channels in the device have a cross-section of 5 × 5 µm^2^. We observed that neutrophils preferred to migrate to alternate branches throughout the network (Supplementary Movie 4). As Fig. 8b shows, six neutrophils arriving at one bifurcation consecutively moved to alternate branches, displaying a pattern of LRLRLR. We measured the fraction of neutrophils moving to alternate branches at each of the 63 bifurcations (Fig. 8c). The fraction is larger than 0.6 at all the bifurcations. The histogram further shows that at more than 87% of the bifurcations, there are larger than 0.7 fraction of neutrophils moving to alternate branches (Fig. 8d). Taken together, our results indicate that neutrophils not only follow this alternate migration pattern in simple bifurcations, but also in complex, interwoven networks of channels.

## Discussion

We investigated the migration pattern of multi-neutrophil squads through branching capillaries in vivo and through microfluidic bifurcations in vitro. We found that consecutively migrating neutrophils in squads preferably enter alternative branches. The in vivo and in vitro models employed in this study share several key features. The geometries of the microfluidic bifurcations and networks resemble the in vivo capillary junctions and networks^4,11–13^. The cross-section of the microfluidic channels (15–50 µm^2^) was within the range of mouse capillaries (~7–100 µm^2^)^11,14^. The match between the in vivo and in vitro data suggests potentially robust mechanisms that guide the alternating migration pattern at non-flow capillary junctions.

The extravasation, infiltration, and trafficking of neutrophils through interstitial space upon tissue injury and infection have been well studied^15^. Moreover, neutrophils also migrate through small capillary networks in organs and tissues such as liver^3^, lymph nodes^5^, skin^4^, and lungs^6,7^. However, how neutrophils efficiently navigate as a group through capillary networks and interstitial spaces is not well understood. Here, we found that the interplay between consecutive neutrophils plays a pivotal role in diverging their migratory path at capillary bifurcations. The process was often neglected in previous studies^16–18^, and may be important for reducing the “traffic jam” in capillary networks. Some of the principles of neutrophil traffic through capillary networks may apply to the traffic through cell-rich tissues that confine the moving neutrophils and limit their directional choices.

Recent reports have shown that neutrophils could attract other neutrophils through LTB4 and other mediators^19–21^. They could also guide T-cell migration by leaving long-lasting chemokine-containing trails^22^. Based on these mechanisms, the it would be expected that the first neutrophil migrating into one branch would attract the second neutrophil to the same branch. However, our results show that neutrophils do not follow each other at bifurcations, which suggesting that the hierarchy of the different neutrophil–neutrophil coordination mechanisms depend on the circumstances. Near large microbial targets neutrophils would follow each other, whereas at a distance from the targets and in conditions of sterile inflammation, the neutrophils will distribute uniformly across patent trafficking paths.

One mechanism responsible for the neutrophils in multi-neutrophil squads entering alternating branches at bifurcations involves the perturbation of chemoattractant gradients behind neutrophils confined and moving in small channels. While the first neutrophil blocks the diffusion of chemoattractant from the anterograde source, the retrograde concentration of chemoattractant in the branch with the moving neutrophil can decrease below the concentration in the alternative branch. The trailing neutrophil follows the higher concentration and enters the unoccupied branch. This mechanism is consistent with the known ability of human neutrophils in channels to steer effectively in the direction of steeper chemoattractant concentrations^23^. The chemical gradients in channels are sensitive to the presence of sustained fluid flow through the channels. Thus, it is important to note that there is no flow in the microfluidic bifurcations in vitro and there is limited or no blood flow in the small capillary junctions obstructed by neutrophils in vivo.

A second mechanism involves the increased hydraulic resistance of the channels due to the presence of neutrophils. The higher hydraulic resistance of neutrophil-occupied channels can also bias the trajectory of the trailing neutrophils towards the unoccupied channels that have lower hydraulic resistance. This bias is consistent with recent studies that showed that neutrophils moving through channels may respond to differences in hydrostatic pressure^9^. This study also offers additional insights into the balance between chemical gradients and hydraulic resistance. Our observations that the alternation between branches at asymmetric bifurcations still takes place when neutrophils did not completely block the larger channels suggests that that the alteration of the chemical gradients may be sufficient to bias neutrophil trajectories. The occasional bias of the second neutrophil toward a higher hydraulic resistance branch is also consistent with the dominant effect of chemical gradients over the hydraulic resistance^9^.

Other strategies that guide the traffic of neutrophils through capillary networks may also be possible. Contact guidance by the side wall of the channel has been proposed for cancer cells based on observations of their migration on printed collagen I lines and bifurcations^24^. Here, we avoided the effect of contact by designing the channel before the bifurcation to be 5 × 5 µm^2^ such that neutrophils contact all channel walls, by their entire circumference. Physical contact between cells has been proposed as a mechanism that alters the trajectory of chemotaxing cancer cells^25^. Here, we observed pairs of neutrophils that maintain contact during migration in channels and found that most “trailing” neutrophils pick alternate branches at bifurcations. These results suggest that the physical contact between neutrophils does not play a role in their alternating trajectories. A mechanism involving water transport through the cell membrane has been proposed to play critical roles in regulating cell migration, including through the confinement of channels^26,27^. Our observations of RBCs and microparticles moving in front of neutrophils at comparable speed with the neutrophils, suggest that unlike the cancer cells, neutrophils are effective at pushing the fluid in front of them. One must note that the average speed of migrating neutrophils in a confined channel is ~20 µm/min, one order of magnitude faster than cancer cells (~50 µm per h)^28^. It is still possible that for neutrophils, the water flux through aquaporins is significantly smaller than the volume of fluid pushed forward by the fast-moving neutrophils, which explains the sensitivity to the hydraulic resistance of the channel in front of the moving neutrophils.

In summary, our study reveals that when marching through complicated capillary networks, neutrophils undertake alternate paths. Chemical and physical mechanisms help distribute the neutrophil traffic uniformly at bifurcations. These mechanisms may contribute to the efficiency of trafficking during infections and inflammation and may help guard against disseminating bacteria.

## Methods

### Device design and fabrication

The microfluidic devices were fabricated with soft lithography. We fabricated the 2-layer master mold in negative photoresist (SU-8, Microchem, Newton, MA) on a 4-in. silicon wafer. The first layer was 5 µm thin which consists of migration channels. The second layer was 150 µm thick which consists of cell-loading channels and chemoattractant chambers. A mixture of PDMS base and curing agent (10:1) (PDMS, Sylgard, 184, Elsworth Adhesives, Wilmington, MA) was cast on the wafer and cured at 65 °C overnight. After overnight curing, we peeled and diced the PDMS layer into individual devices. We punched the inlets and outlets of the devices using a 0.75 mm diameter biopsy puncher (Harris Uni-Core, Ted Pella) and irreversibly bonded them to a glass-bottom multiwell plate (MatTek Co., Ashland, MA).

### Multiphoton microscopy in vivo

Animal experiments were performed with male and female adult mice (6–10 week old), and all experimental animal protocols were approved by the University of Calgary Animal Care Committee and were in compliance with the Canadian Council for Animal Care Guidelines. Multiphoton microscopy was employed to assess the movement and location of neutrophils in lymph node capillaries following infection with *S. aureus*. 2.5 × 10^7^ CFU were injected into footpad before imaging. Mouse popliteal lymph nodes were imaged by anesthetizing the mouse and exposing the popliteal lymph node in the right hind-limb. Anesthetic and TRITC-Dextran 70,000 kDa were delivered by cannulation of right jugular vein. Image acquisition of the popliteal lymph node was performed an upright multiphoton microscope (Olympus FV1000 MPE, Richmond Hill, Ontario, Canada). Neutrophils and vasculature were visualized simultaneously in separate channels using 830 nm pulsed laser Ti:sapphire excitation (Coherent Chameleon Ultra II). The fluorescence emission was directed through bandpass filters (GFP: 520 ± 20 nm and TRITC: 600 ± 30 nm) and detected by non-descanned photomultiplier detectors (Olympus). Fluoview (FV10-ASW4.2) software was used to drive the confocal microscope and for 3D rendering of images. Files in OIF format were imported into Fiji (7) for analysis or Volocity 6.3 (Perkin Elmer) for export.

### Spinning-disk confocal intravital microscopy

A tail vein catheter was inserted into mice after anesthetization with 200 mg/kg ketamine (Bayer Animal Health) and 10 mg/kg xylazine (Bimeda-MTC). Sterile inflammation was performed as described previously^3^. In brief, mice were anesthetized with isoflurane and a <1-cm incision was made just below the level of the diaphragm to expose the liver. A single focal injury was induced on the surface of the liver using the tip of a heated 30-gauge needle mounted on an electro-cautery device. Mice were prepared for intravital microscopy of the liver as previously described^3,13,29^. Briefly, a midline laparotomy was performed followed by removal of the skin and abdominal muscle along the costal margin to the midaxillary line to expose the liver. Mice were placed in the right lateral position and a single liver lobe was exteriorized on the pedestal of a custom-made heat controllable Plexiglas microscope stage. All exposed tissues were moistened with saline-soaked gauze to prevent dehydration during imaging. For the duration of all experiments, the liver was continuously superfused with physiological saline buffer. Images were acquired using Olympus IX81 inverted microscope, equipped with an Olympus focus drive and a motorized stage (Applied Scientific Instrumentation, Eugene, OR) and fitted with a motorized objective turret equipped with 10×/0.40 UPLANSAPO, and 20×/0.70 UPLANSAPO objective lenses and coupled to a confocal light path (WaveFx; Quorum Technologies, Guelph, ON) based on a modified Yokogawa CSU-10 head (Yokogawa Electric Corporation, Tokyo, Japan). The hepatic microvasculature and neutrophils were visualized by intravenous (i.v.) infusion of 1.2 mg of PE- conjugated anti-PECAM-1 and APC-conjugated anti-Ly6G antibodies, respectively. We assume a capillary has a circular cross-section and estimate the cross-sectional area of the capillary as *π*(*w*/2)^2^, where *w* is the width of the capillary. Laser excitation wavelengths of 491, 561, and 642 nm (Cobolt) were used in rapid succession together with the appropriate band-pass filters (Semrock). A back-thinned EMCCD 512 × 512 pixel camera was used for fluorescence detection. Volocity software (Perkin Elmer) was used to drive the confocal microscope.

### Neutrophil isolation

Human blood samples from healthy donors (aged 18 years and older) were purchased from Research Blood Components, LLC (Brighton, MA). Human neutrophils were isolated within 2 h after drawn using the human neutrophil direct isolation kit (STEMcell Technologies, Vancouver, Canada). Isolated neutrophils were stained with Hoechst 33342 trihydrochloride dye (Life Technologies) and then suspended in Iscove’s Modified Dulbecco’s Medium (IMDM) containing 20% fetal bovine serum (FBS) (Thermo Fisher Scientific) at a concentration of 1 × 10^7^ cells per mL.

### Microfluidic device operation and imaging

Formulated peptide fMLP (Sigma-Aldrich) was diluted in IMDM containing 20% FBS to 100 nM. To prime the device, 10 µL of the chemoattractant solution was pipetted into each device. The well plate was then placed in a desiccator under vacuum for 10 min and then taken out for 15 min until the devices were filled completely with the solution. Three microlitre of media (IMDM + 20% FBS) was then added to each well to cover the devices. Ten microlitre media was then pipetted from the inlet to replace the chemoattractant in the cell-loading channel with chemoattractant-free media which created the gradient along the migration channels. Two microlitre neutrophil suspension was then pipetted into each device. To characterize the chemoattractant gradient, the fMLP solution was spiked with fluorescein (Sigma-Aldrich) at a concentration of 1 µg/mL and loaded in the device. To characterize the movement of fluid in the channels, RBCs (~2 × 10^8^ per mL) or 2 µm nile-red polystyrene microbead (1.04 g per mL, 0.1% w per v) (Spherotech Inc., Lake Forest, IL, USA) were spiked into the fMLP solution and loaded in the device.

Time-lapse images at regions of interest were captured at 10× or 20× magnification with a time interval from 30 s to 3 min between two cycles, using a fully automated Nikon TiE microscope (Micro Device Instruments). The microscope is equipped with a biochamber heated at 37 °C and 5% CO_2_. The trajectories of cells and microbeads as well as the fluorescent intensity profiles were analyzed using Fiji ImageJ.

### Estimating fluid displacement in channels by neutrophils

To estimate the fluid displacement in front of moving neutrophils, we employed floating microparticles (fresh, human RBCs, or polystyrene microbeads). We loaded the channels with a small number of microparticles during the device-priming steps. We identified neutrophils moving in channels inside which one or more microparticles were present. We measured the change in the position of microparticles that were at 50 µm or more in front of moving neutrophils. Whenever the microparticles moved with velocities comparable to those of the moving neutrophils, we concluded that fluid flow is present in front of the moving neutrophils. We only made qualitative estimates regarding the displacement of fluid in front of moving neutrophils because several factors may affect the microparticle velocity and preclude the use of microparticles for quantitative measurements of fluid flow. For example, the velocity of RBCs in the centerline of a ~10 µm diameter channel during Poiseuille flow can be up to ~1.5 faster than the average velocity of the fluid in the channel^30^, an effect known as the Fahraeus effect^31^. Other factors, including the size of the microparticle relative to the size of the channel, the friction between the microparticle and the walls of the channel could reduce the velocity of particles below that of the average velocity of the fluid. Consequently, or analysis of the microparticle and neutrophil velocities could only identify if fluid is being displaced inside small channels during neutrophil migration.

### Reporting summary

Further information on research design is available in the Nature Research Reporting Summary linked to this article.

## Supplementary information

Supplementary Information

Description of Additional Supplementary Files

Supplementary Movie 1

Supplementary Movie 2

Supplementary Movie 3

Supplementary Movie 4

Reporting Summary

## Data Availability

The authors declare that the data are available in the paper (and its Supplementary Information files) or from the corresponding author upon reasonable request. The raw data underlying Figs. 2–7 are provided as a Source Data file and are also accessible in Figshare: 10.6084/m9.figshare.9971426.v1.

## Source data

Source Data
