# Supplementary material for: Chemotaxing neutrophils enter alternate branches at capillary bifurcations
Source: Nat Commun. 2020 May 13;11:2385. doi: 10.1038/s41467-020-15476-6 (PMC7220926; doi:10.1038/s41467-020-15476-6)
Supplement: Supplementary file 1 — Supplementary Information [file 41467_2020_15476_MOESM1_ESM.pdf]

Supplementary information

Chemotaxing Neutrophils Enter Alternative Branches at Capillary Bifurcations

Xiao Wang, Mokarram Hossain, Ania Bogoslowski, Paul Kubes, and Daniel Irimia

Supplementary Figure 1. Chemoattractant gradients at bifurcations.

Supplementary Figure 2. Outline of bifurcations analyzed in movie.

Supplementary Figure 3. Neutrophil bias at symmetric and asymmetric bifurcations of various sizes.

Supplementary Figure 4. Fluorescent-intensity gradients at bifurcations.

Supplementary Table 1. Quantitative analysis of two-neutrophil decision making at capillary bifurcations *in vivo*.

Supplementary Movie 1 In vivo imaging of neutrophil trafficking in mouse liver. Highlighted areas identify neutrophil pairs arriving at capillary bifurcations, following liver tissue injury.

Supplementary Movie 2 In vivo imaging of neutrophil trafficking in mouse lymph nodes. Highlighted areas identify neutrophil pairs arriving at capillary bifurcations, following infections with Staphylococcus aureus.

Supplementary Movie 3 Neutrophil squads migrating through microfluidic bifurcations enter alternative branches. Neutrophils are stained in blue using Hoechst dye.

Supplementary Movie 4 A large group of neutrophils migrating through microfluidic bifurcation networks also enter alternative branches at bifurcations. Overview and zoom-in at one location.

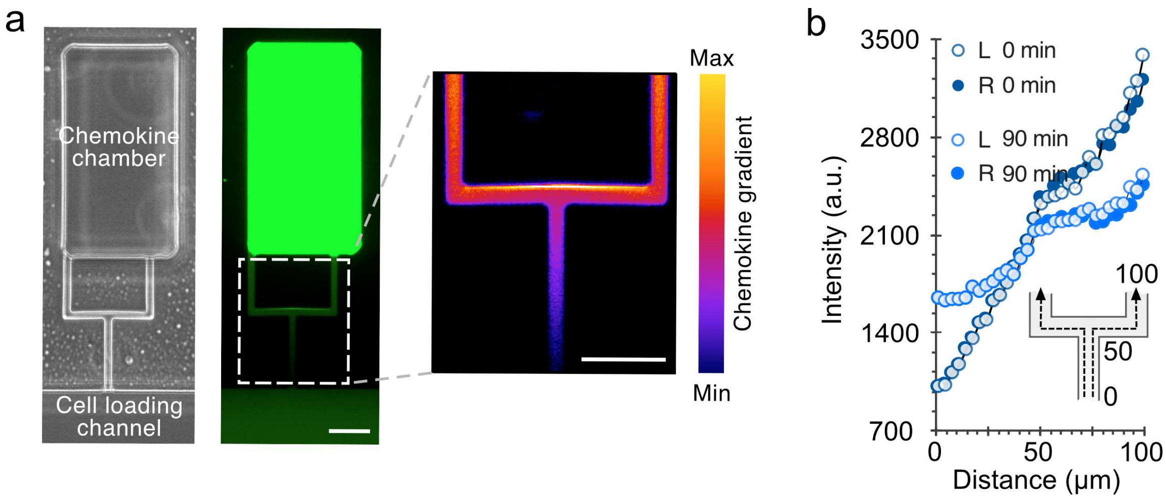

Supplementary Figure 1. Chemoattractant gradients at bifurcations (a) Bright-field and fluorescent microscopic images showing the chemoattractant gradients at bifurcations. The zoom-in panel is pseudo-colored fire to highlight the gradient along the channels. The scale bar is 25μm. (b) The measurements of the fluorescent profile along the left and right bifurcations at 0 and 90min. The dark blue hollow and solid dots represent left and right branches at 0 min. The light blue hollow and solid dots represent left and right branches at 90 min.

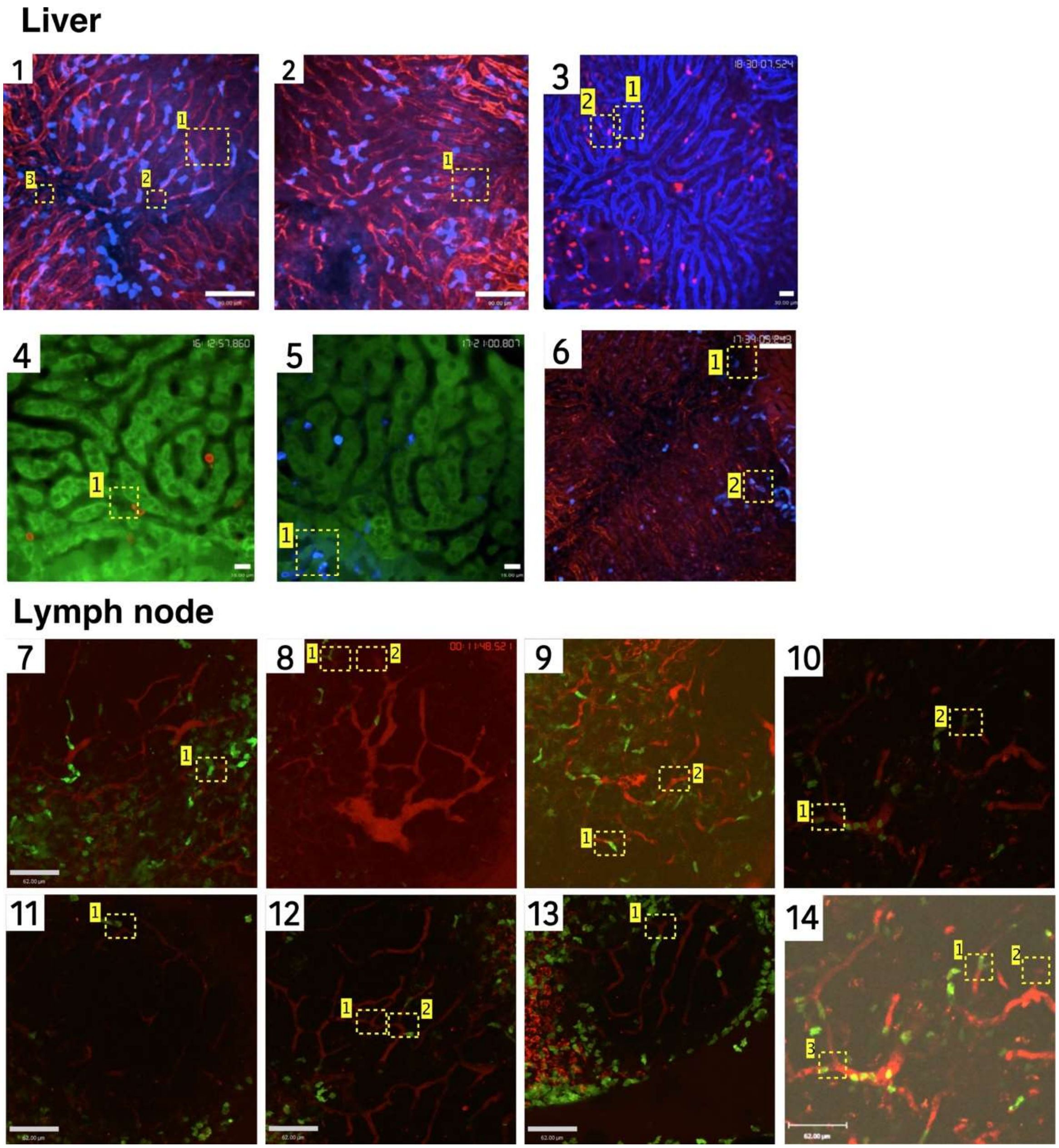

Supplementary Figure 2. Outline of bifurcations analyzed in movie. Locations where more than two neutrophils migrated through capillary bifurcations consecutively in the section 1-6 (liver, N=4 mice) in the supplementary video 1 and section 7-14 (lymph node, N=8) in the supplementary video 2. The yellow dashed squares in the panel 1-14 highlight the locations in the corresponding sections.

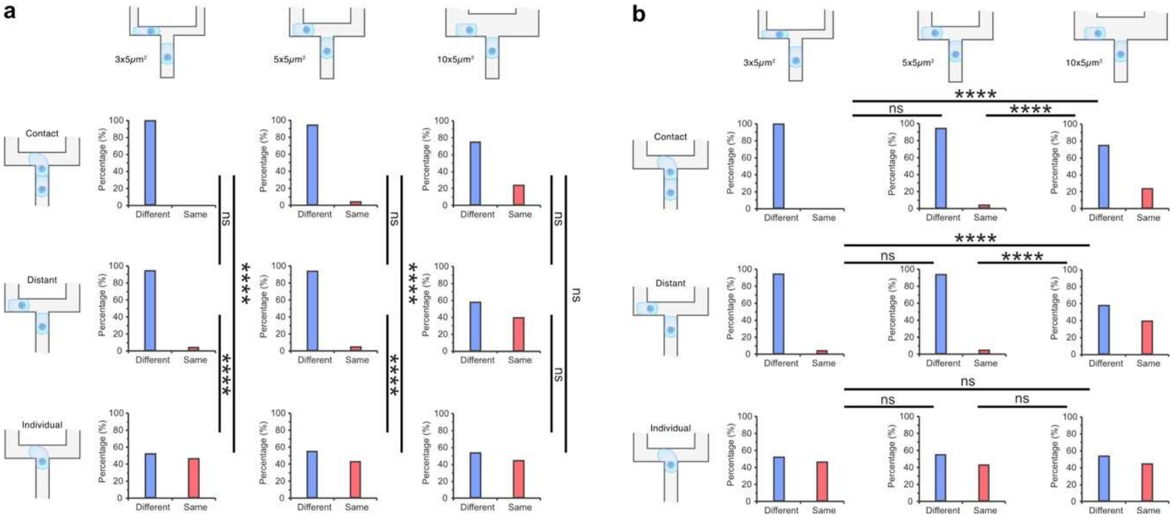

Supplementary Figure 3. Neutrophil bias at symmetric and asymmetric bifurcations of various sizes. Statistical analysis of the differences between the percentages of neutrophils moving to same or different branches for (a) 3 categories of intercellular distances and (b) 3 channel cross-sections (N=3, N=20-100) (**** *p < 0.0001*, Fisher's exact test).

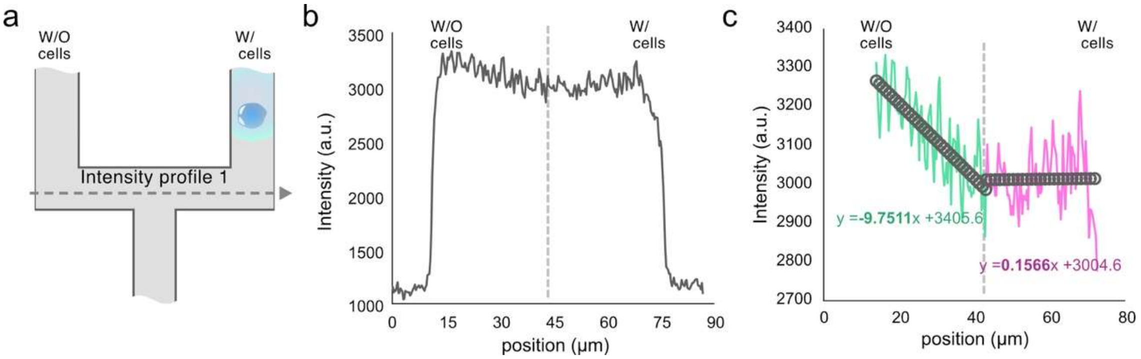

Supplementary Figure 4. Fluorescent-intensity gradients at bifurcations. **(**a) a schematic showing the measurement the measurement location of the fluorescence intensity profile in the bifurcation channel. A neutrophil is migrating in one of the branches. (b) Fluorescent intensity profile along the intensity profile 1 in (a). The grey dashed line indicates splitting location of two branches. (c) Measurements of fluorescent gradient in the two branches along profile 1. The green and purple lines represent fluorescent profiles in the branches without and with the neutrophil, respectively. The circled lines are the linear fit of the fluorescent profiles. The absolute value of the slope of the fit curve is the fluorescent intensity gradient.

Supplementary Table 1. Quantitative analysis of two-neutrophil decision making at capillary bifurcations *in vivo*. The bifurcations where the capillary branches are both smaller than 50 μm^2 are highlighted in red. The size of each branch is presented (μm^2). Data from liver (N=4 mice) and lymph nodes (N=8 mice).

**Section**	**Sample**	**Size of branches (μm^2)**	**Location**	**# of events**	**Same or different branches**		
**1**	Liver, local damage	29.2/58.5	1	3	D	S	S
		31.5/23.7	2	1	D		
		113.0/69.3	3	1	D		
**2**	Liver, local damage	100.0/85.9	1	1	D		
**3**	Liver, local damage	51.5/120.7	1	1	D		
		47.7/35.2	2	1	D		
**4**	Liver, local damage	56.7/59.4	1	1	D		
**5**	Liver, local damage	NA/NA	1	1	D		
		NA/NA	1	1	S		
**6**	Liver, local damage	52.8/9.6	1	1	S		
		54.5/52.6	2	1	S		
**7**	Lymph node, *S aureus* infection	39.6/41.2	1	1	S		
**8**	Lymph node, *S aureus* infection	40.1/15.6	1	1	D		
		7.2/7.4	2	1	D		
**9**	Lymph node, *S aureus* infection	12.9/16.8	1	1	D		
		27.0/21.3	2	1	D		
**10**	Lymph node,	77.7/27.7	1	1	D		

	S aureus infection	19.1/21.1	2	1	D		
**11**	Lymph node, *S aureus* infection	7.8/12.2	1	1	D		
**12**	Lymph node, *S aureus* infection	8.0/23.6	1	1	S		
		16.5/30.3	2	1	D		
**13**	Lymph node, *S aureus* infection	8.5/16.6	1	2	S	S	
**14**	Lymph node, *S aureus* infection	28.3/12.6	1	2	D	D	
		38.5/19.6	2	2	D	D	
		41.8/21.6	3	3	D	D	D
