# Supplementary material for: Chemotaxing neutrophils enter alternate branches at capillary bifurcations
Source: Nat Commun. 2020 May 13;11:2385. doi: 10.1038/s41467-020-15476-6 (PMC7220926; doi:10.1038/s41467-020-15476-6)
Supplement: Supplementary file 2 — Description of Additional Supplementary Files [file 41467_2020_15476_MOESM2_ESM.pdf]

Description of Additional Supplementary Files

File Name: Supplementary Movie 1
Description: In vivo imaging of neutrophil trafficking in mouse liver. Highlighted areas identify neutrophil pairs arriving at capillary bifurcations, following liver tissue injury.

File Name: Supplementary Movie 2
Description: In vivo imaging of neutrophil trafficking in mouse lymph nodes. Highlighted areas identify neutrophil pairs arriving at capillary bifurcations, following infections with Staphylococcus aureus.

File Name: Supplementary Movie 3
Description: Neutrophil squads migrating through microfluidic bifurcations enter alternative branches. Neutrophils are stained in blue using Hoechst dye.

File Name: Supplementary Movie 4
Description: A large group of neutrophils migrating through microfluidic bifurcation networks also enter alternative branches at bifurcations. Overview and zoom-in at one location.
